# Supplementary material for: Protection by -Biotics against Hypertension Programmed by Maternal High Fructose Diet: Rectification of Dysregulated Expression of Short-Chain Fatty Acid Receptors in the Hypothalamic Paraventricular Nucleus of Adult Offspring
Source: Nutrients. 2022 Oct 14;14(20):4306. doi: 10.3390/nu14204306 (PMC9609147; doi:10.3390/nu14204306)
Supplement: Supplementary file 1 [file nutrients-14-04306-s001.zip › nutrients-1954926-supplementary.pdf]

Protection by -Biotics against Hypertension Programmed by Maternal High Fructose Diet: Rectification of Dysregulated Expression of Short-Chain Fatty acid Receptors in the Hypothalamic Paraventricular Nucleus of Adult Offspring

Yung Mei Chao, You Lin Tain, Wei-Chia Lee, Kay L.H. Wu, Hong-Ren Yu
and Julie Y.H. Chan

Supplementary Figure S1: Oral supplements with –biotics have no significant effect on tissue ROS production and proinflammatory cytokines release in the PVN of ND offspring.

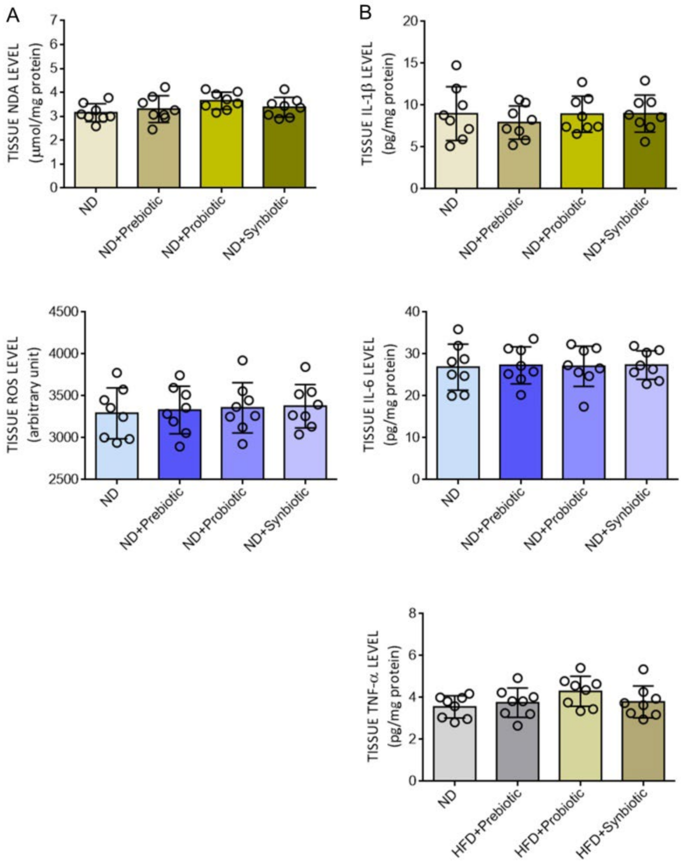

Figure S1: Tissue malondialdehyde (MDA) and reactive oxygen species (ROS) levels (**A**), and levels of proinflammatory cytokines interleukin 1-β (IL-1β), interleukin 6 (IL-6), and tumor necrosis factor α (TNF-α) (**B**) in the hypothalamic paraventricular nucleus (PVN) of adult ND offspring that received oral supplementation of FOS (4 g/kg/day, probiotic), *L. gasseri* (3×10^6 CFU/mL/day, probiotic) or FOS+*L. gasseri* (synbiotic) initiated at age of 6 weeks. Data are presented as mean ± SD, $n = 8$ in each group. No significant difference in one way ANOVA among various groups.

Supplementary Figure S2: Oral supplements with –biotics restores plasma butyrate level in adult HFD offspring.

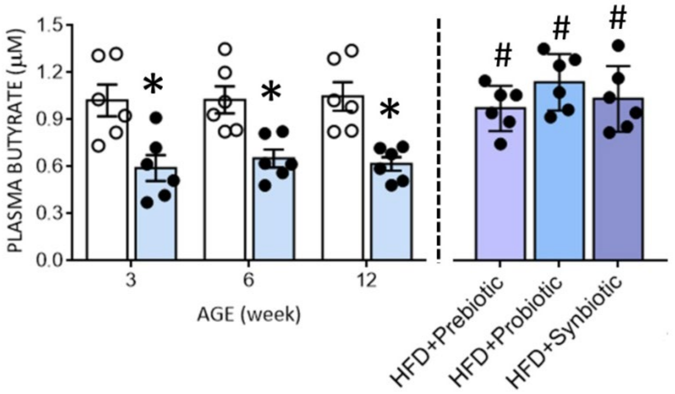

Figure S2. Plasma butyrate levels of ND (open circles) or HFD (filled circles) offspring at the age of 3, 6 and 12 weeks, or at 12 weeks after additional oral supplementation of FOS (4 g/kg/day, probiotic), *L. gasseri* (3 × 10^6 CFU/mL/day, probiotic) or FOS+*L. gasseri* (synbiotic) initiated at the age of 6 weeks. Data are presented as mean ± SD, $n = 6$ in each group. *$P < 0.05$ vs. ND group, and $^{\#}P < 0.05$ vs. HFD group in the post hoc Tucky's multiple-range test.
